# Unusual Case of an Idiopathic Thyrocervical Trunk Pseudoaneurysm: An Interesting Case Report

**DOI:** 10.7759/cureus.72273

**Published:** 2024-10-24

**Authors:** Rubén Neris, Elmer R De Camps Martinez, Elizabeth Leon, Nelson Encarnacion

**Affiliations:** 1 General Surgery, Trumbull Regional Medical Center, Warren, USA; 2 Internal Medicine, Trumbull Regional Medical Center, Warren, USA; 3 Internal Medicine, Hospital Plaza de la Salud, Santo Domingo, DOM; 4 Vascular Surgery, Hospital Plaza de la Salud, Santo Domingo, DOM

**Keywords:** cardiology, open approach, pseudoaneurysm, thyrocervical trunk, vascular surgery

## Abstract

Thyrocervical trunk pseudoaneurysms are very rare and usually occur after trauma or invasive procedures. Given its location and nature, thyrocervical trunk pseudoaneurysm typically presents with symptoms at presentation. Those that occur spontaneously and without symptoms are even more uncommon. The management of thyrocervical trunk pseudoaneurysm can be done in three main ways: endovascular, open, or hybrid (endovascular and open).

This case report describes a 71-year-old male Hispanic patient who presented to the vascular surgery clinic with a left supraclavicular mass that he noticed 12 years ago. There was no significant past medical history or history of neck surgery/interventions. Besides the pulsatile mass, the patient did not endorse any other complaints. A duplex ultrasound (DUS) was obtained, and it was consistent with a pseudoaneurysm. A neck computed tomography angiogram (CTA) was subsequently obtained and confirmed the large (6.1×4.6×5 cm) pseudoaneurysm at the root of the left thyrocervical trunk. The decision was made to take him to the operating room. Using an open approach, the left thyrocervical trunk was dissected, and the pseudoaneurysm was exposed. The pseudoaneurysm was then resected, and a primary anastomosis of the vessel was performed.

The patient tolerated the procedure well without complications. He was discharged on post-operative day 2 in stable condition. Short-term follow-up demonstrated no left supraclavicular mass recurrence on physical examination. The surgical incision is healing well.

Thyrocervical trunk pseudoaneurysms are rare entities that can be detrimental to the patient if not treated optimally. Even if the patient remains asymptomatic or relatively asymptomatic, the incidence of embolization, thrombosis, or rupture is high. Open vascular intervention is recommended when the root of the trunk is involved.

## Introduction

The thyrocervical trunk is the second superior branch of the subclavian artery. Given its location, deep in the neck zone I, it is well-protected from blunt trauma [1,2]. Out of all the peripheral artery pseudoaneurysms, the ones located at the upper extremity are the rarest in incidence. Among these, the subclavian artery is the most common, followed by the thyrocervical trunk [3]. The vast majority of thyrocervical trunk pseudoaneurysms are iatrogenic, following attempted central venous catheter or temporary hemodialysis catheter placement in the internal jugular or subclavian vein. Common non-iatrogenic causes of thyrocervical trunk pseudoaneurysm include penetrating trauma, such as a gunshot wound or stab wound [4,5]. The most common symptom of a thyrocervical trunk pseudoaneurysm is a pulsatile mass with bruit [6]. However, complications may arise from the pseudoaneurysm, including pain, expanding pulsatile mass, distal embolization, thrombosis, local compression, and transient cerebral ischemia [7]. Pseudoaneurysms, including the thyrocervical trunk, normally require intervention because of the risk of associated complications, such as rupture, mass effect, and pain [8].

The diagnosis of a thyrocervical trunk pseudoaneurysm begins with a detailed history and physical examination. As described above, the symptoms and complications are usually typical in these patients. The recommended approach in confirming the suspected diagnosis of a thyrocervical trunk pseudoaneurysm includes duplex ultrasound (DUS), computed tomography angiogram (CTA), or arteriography. Given its complete definition and details, a digital subtraction angiography (DSA) or formal arteriography is often necessary if the diagnosis is still debatable [9].

In the past, most of the thyrocervical trunk pseudoaneurysms have been managed with an open surgical approach. More recently, the endovascular approach has become more frequent and often used as the first-line treatment. However, many vascular surgeons worldwide prefer to perform an open-approach repair when the location of the pseudoaneurysm is at the root of the trunk. In these situations, the ideal treatment option is debatable, and the approach should be dictated by the surgeon's preference. There is also a hybrid approach, which includes both open and endovascular, used in selected cases [10].

This case report describes a case of a large, idiopathic thyrocervical trunk pseudoaneurysm at the root of the trunk with a pulsatile mass as the only symptom. An open surgical approach was performed with a successful resection of the pseudoaneurysm.

## Case presentation

A previously healthy 71-year-old Hispanic patient presented to the vascular surgery outpatient clinic referred by his primary care provider with a chief complaint of a painless left supraclavicular pulsatile mass that he has had for the past 12 years but did not seek any medical attention until then. There was no history of trauma or invasive neck procedures reported by the patient. On the review of systems, he denied dysphagia, neck pain, weakness/tingling/numbness of the upper extremities, signs of upper extremity ischemia, or stroke-like symptoms. He denied significant family history, including a history of vasculitis, aneurysm, connective tissue disease, or other vascular diseases. The patient was a former smoker of 29 pack-years but quit 28 years ago. He denied medication intake at the time.

**Figure 1:**
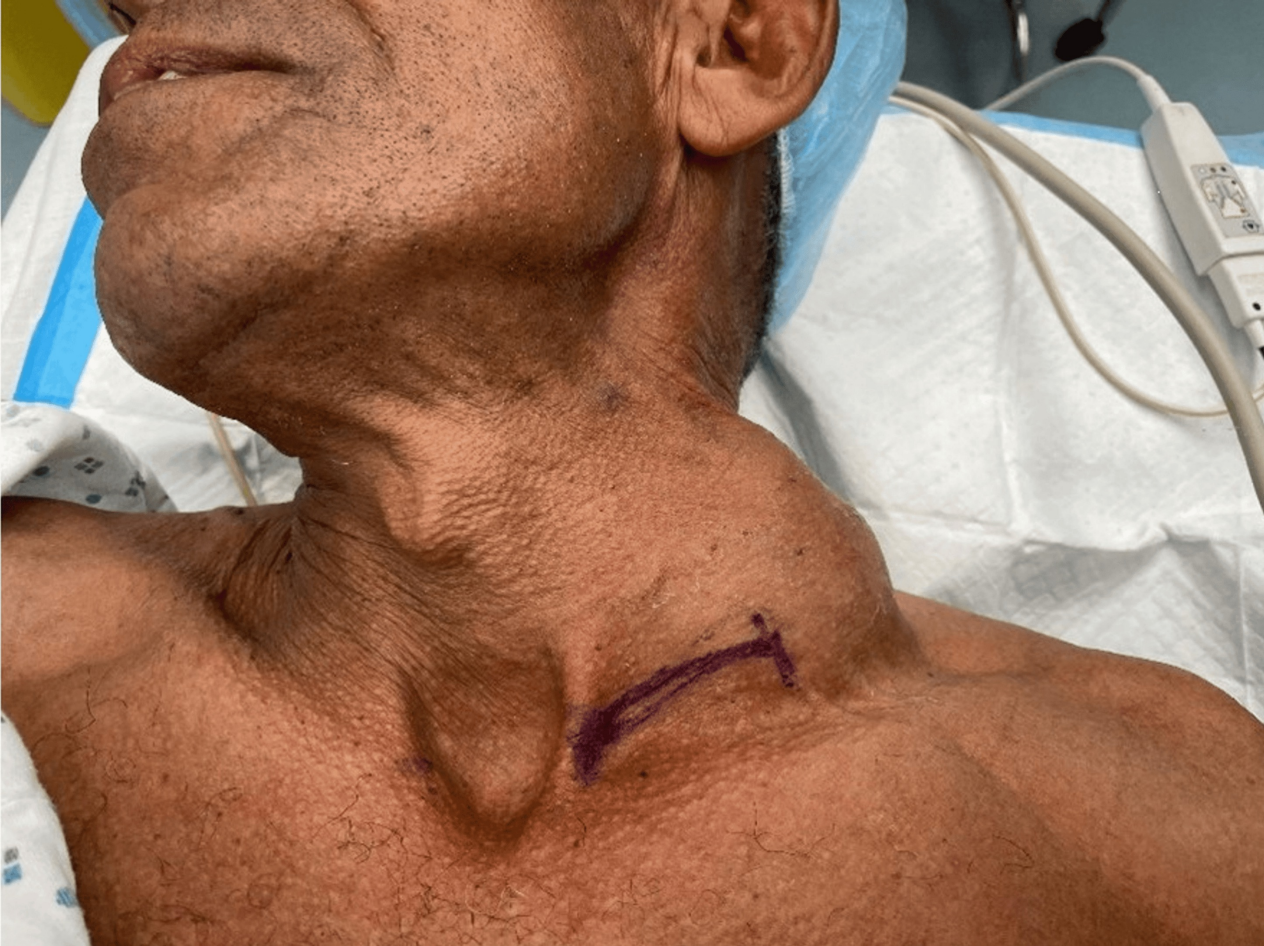
Large, left supraclavicular mass noted. The mass was non-tender and mobile on palpation, associated with a bruit on auscultation. Size measurement: 6×5 cm.

On physical examination, the patient was in no acute distress and did not look ill or toxic. His vital signs were stable. There was a left supraclavicular, non-tender, pulsatile mass (Figure 1) that was mobile on palpation and associated with a bruit on auscultation. An intact neurovascular examination was noted.

**Figure 2:**
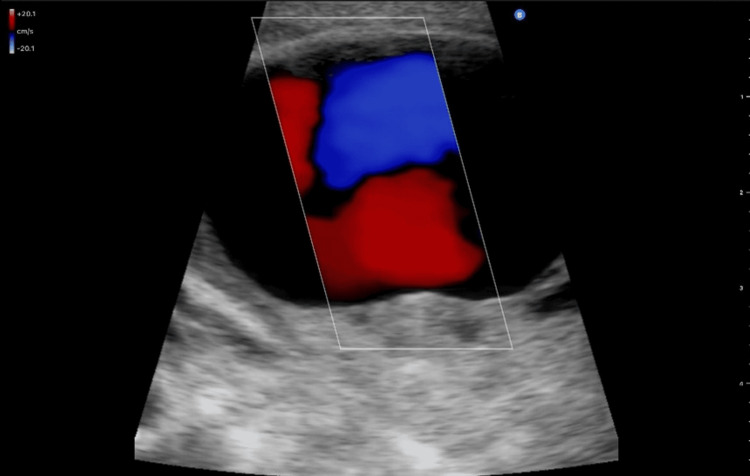
DUS overlying the left supraclavicular mass. Note the bidirectional flow with the "to-and-fro" sign. DUS: duplex ultrasound

Complete blood count, complete metabolic panel, liver function test, and coagulation studies were unremarkable. DUS was initially obtained for the further workup of the left supraclavicular mass and showed bidirectional flow with a "to-and-fro" sign and mural isoecogenic mass within the vessel (Figure 2), suggestive of a pseudoaneurysm.

**Figure 3:**
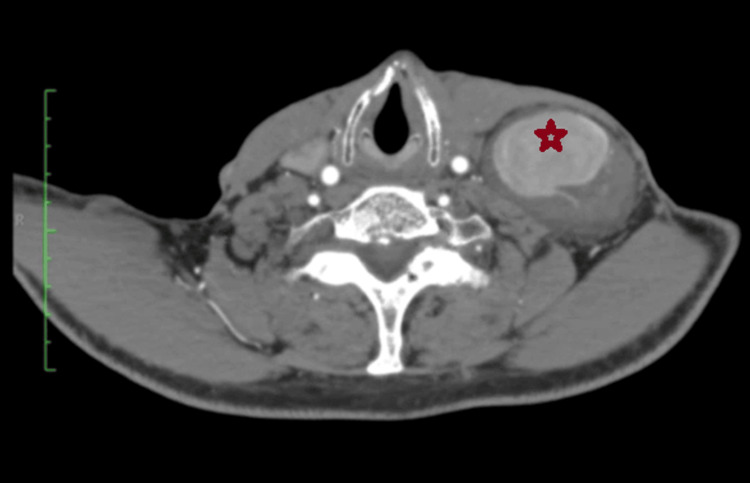
CTA of the chest showing a large, left thyrocervical trunk pseudoaneurysm (at the root) with associated mural thrombus (red star). CTA: computed tomography angiogram

A CTA of the chest was subsequently performed to fully delineate the location of the mass and its characteristics and demonstrated a saccular dilation at the root of the left thyrocervical trunk that measured 6.1×4.6×5 cm (Figure 3). Given the findings of the DUS and the CTA, the diagnosis of a left thyrocervical trunk pseudoaneurysm was confirmed.

**Figure 4:**
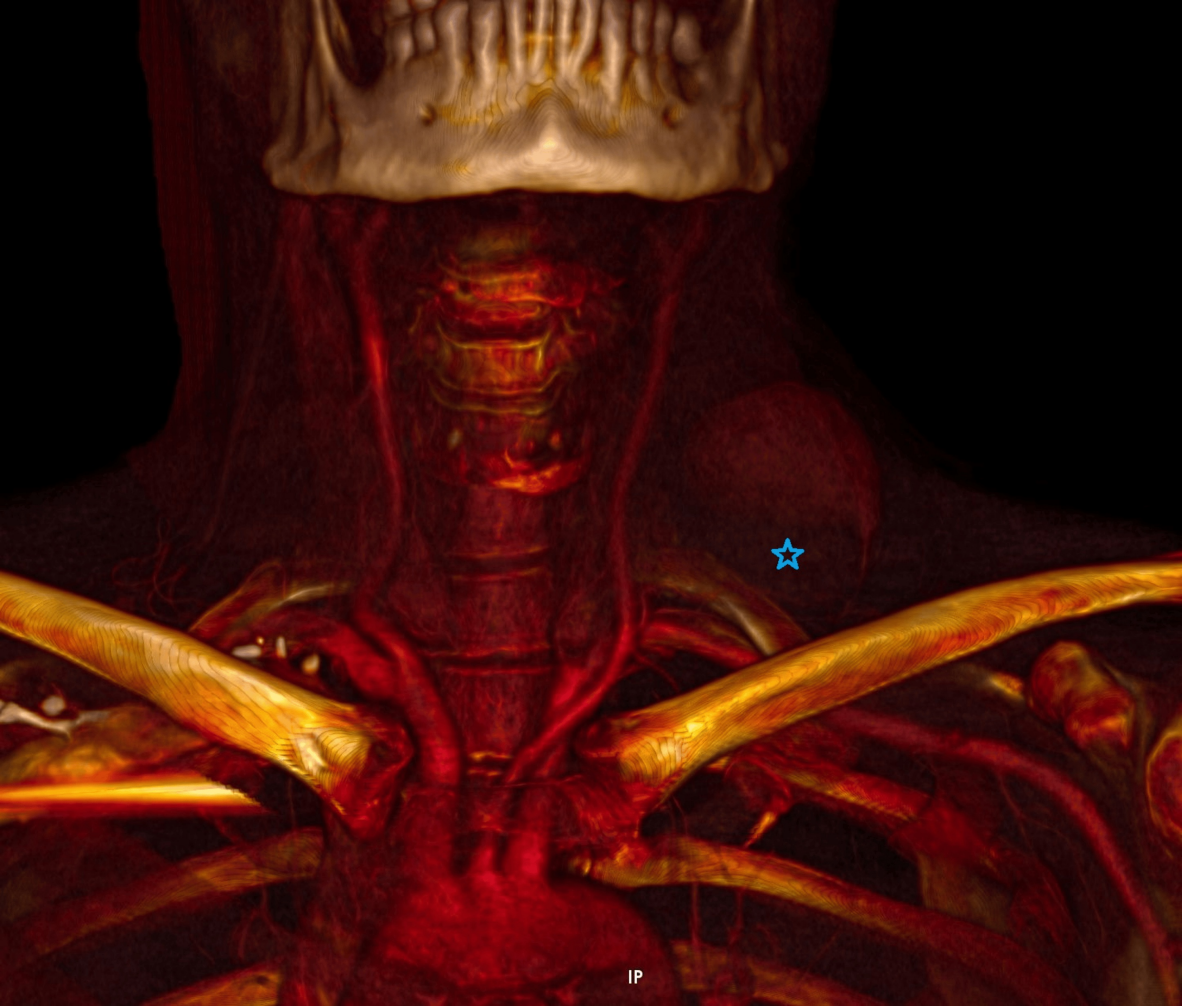
Large, left thyrocervical trunk pseudoaneurysm noted (blue star), this time with the CT 3D reconstruction. CT: computed tomography

A computed tomography (CT) 3D reconstruction of the chest was also obtained for surgical planning and workup completion (Figure 4).

We discussed the endovascular versus open approach with the patient. Given the location of the pseudoaneurysm at the root of the thyrocervical trunk and the significant size, the open approach was elected.

**Figure 5:**
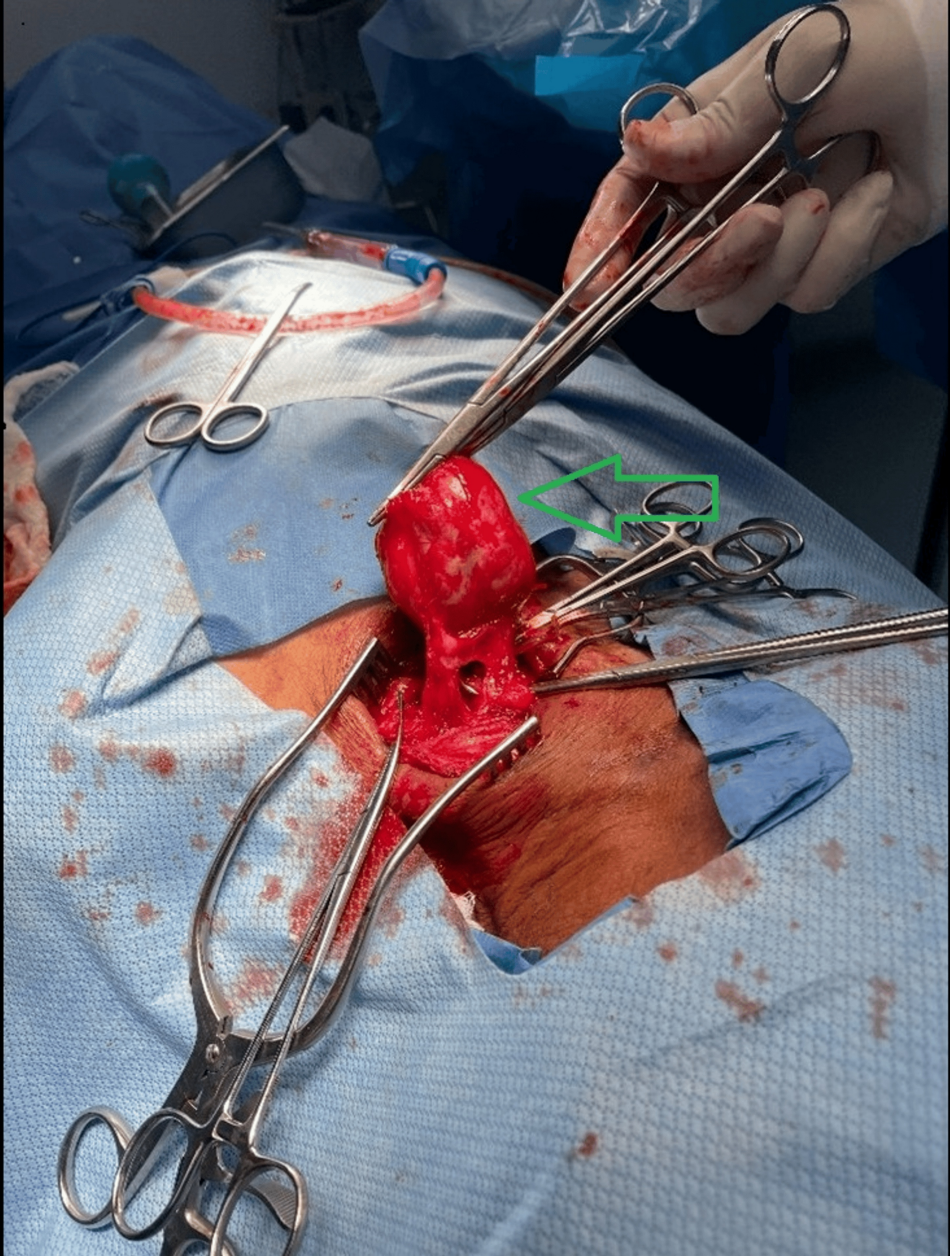
A left neck transverse incision has been made and dissection performed all the way down to the left thyrocervical trunk. The left thyrocervical trunk pseudoaneurysm sac has been dissected from surrounding tissues and grabbed with straight clamps. As shown by the green arrow, the pseudoaneurysm sac is located at the root of the trunk.

The patient was brought to the operating room and placed supine. The neck was mildly extended using a shoulder roll behind the upper back and the neck rotated to the right. The left neck was prepped and draped in the usual sterile fashion. A transverse incision was made at the left supraclavicular region. The dermis, subcutaneous tissue, and muscles were dissected using cautery and blunt dissection. The left subclavian artery was then identified and dissected free from surrounding tissues. Cephalad dissection was then performed, and the left thyrocervical trunk with the pseudoaneurysm located at the root was identified and dissected free from surrounding tissues (Figure 5). Collateral branches of the left thyrocervical trunk were ligated with silk 2-0. The proximal and distal of the artery were obtained using vascular clamps. The pseudoaneurysm was then resected successfully, and a primary anastomosis was done with 6-0 Prolene sutures. The hemostatic agent Surgicel was placed for hemostasis control. A Blake drain was placed, and the incision was closed in a multilayer fashion. The patient tolerated the procedure well and was transferred to the intensive care unit in stable condition.

**Figure 6:**
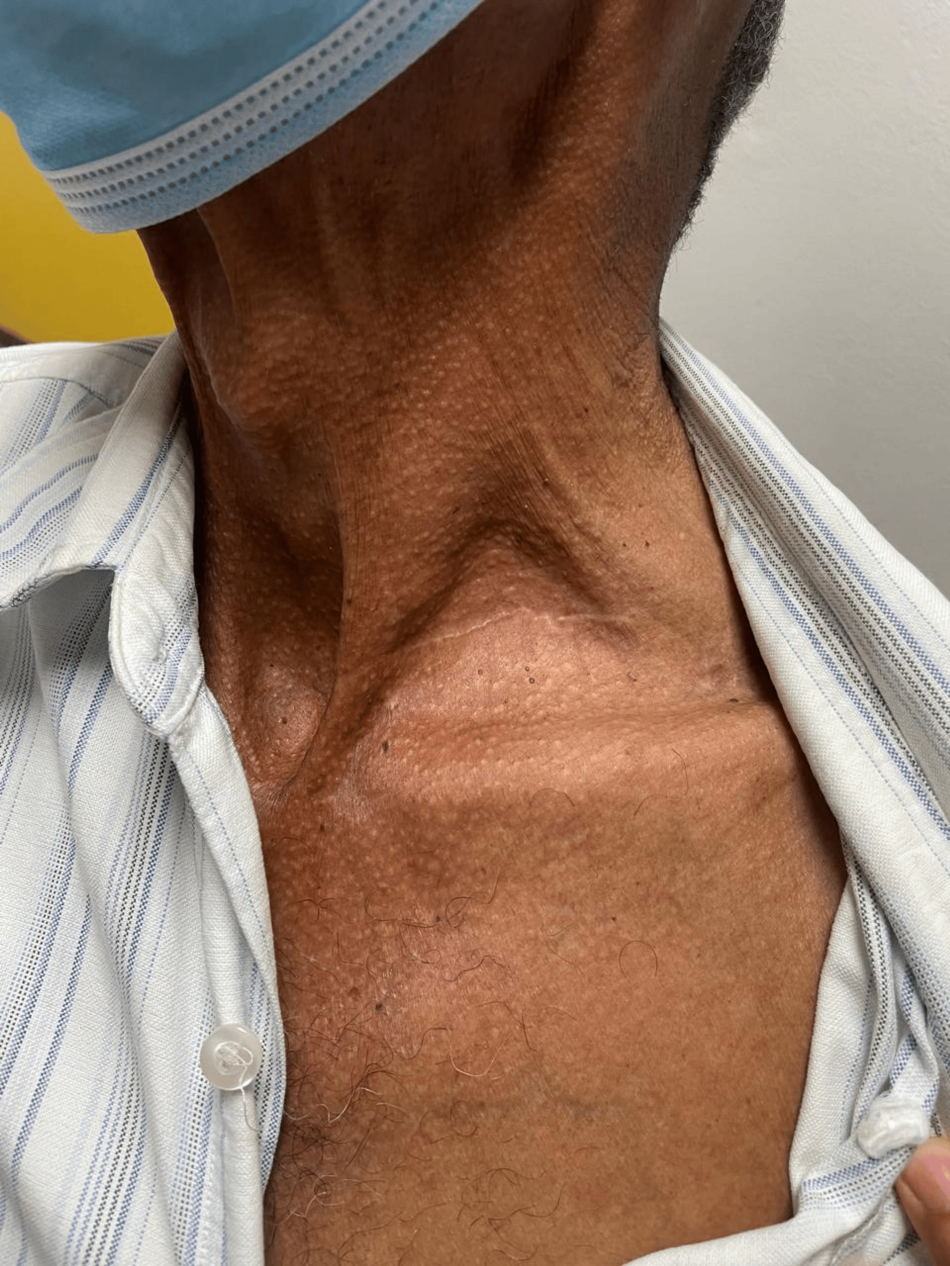
The left neck two weeks post-operatively. No mass or bruit noted. The surgical wound scar healed well.

The patient was discharged on post-operative day 2 without neurological or vascular impairments. The patient was seen in the office for a follow-up two weeks after the surgery. No mass or bruit was detected on his left neck, and the surgical incision was noted to be healing well (Figure 6).

## Discussion

In this case report, we describe a case of a large thyrocervical trunk pseudoaneurysm treated successfully with open surgical repair. In the current literature, pseudoaneurysms are well-documented complications following trauma or iatrogenic injury, but the incidence in the thyrocervical trunk is extremely rare, with the vast majority happening secondary to trauma or iatrogenic in nature (0.5%-1% of all aneurysms) [11]. The incidence of idiopathic thyrocervical trunk aneurysm is even rarer with limited documentation in the literature [12].

The gold standard test for the diagnosis of thyrocervical trunk pseudoaneurysm still remains formal angiography. Angiography demonstrates a more complete definition of the pseudoaneurysm, location and size. However, the DUS can be used initially with good diagnostic accuracy. The DUS has a sensitivity of 94% and a specificity of 97% in the diagnosis of pseudoaneurysm. Moreover, the DUS typically shows the swirling motion in the sac, known as the "to-and-fro" waveform, which is diagnostic [13]. CTA and magnetic resonance angiography (MRA) can be also obtained with equal diagnostic value. It has the advantage of visualizing the surrounding tissues and detecting other possible comorbidities around the pseudoaneurysm. Many surgeons also obtain them for surgical planning [14].

A thyrocervical trunk pseudoaneurysm can be treated in different ways. Available options include open surgical repair, endovascular repair, a hybrid procedure, and ultrasound-guided thrombin injection. Although the ultrasound-guided thrombin injection is frequently used in groin pseudoaneurysms, it is not usually recommended for thyrocervical trunk pseudoaneurysm. It has many disadvantages in this situation, such as the inability to apply in cases of trunk lesions, the inability to obtain hemostasis by itself, and the high risk of systemic thrombosis or embolization. Among the treatment options, the endovascular approach is becoming the most frequent method, given its less invasive nature. When the pseudoaneurysm is located at the root of the trunk, the anatomy of the lesion is more complex, and the repair approach is more controversial. These pseudoaneurysms have been treated historically by an open surgical approach [15].

Thyrocervical trunk pseudoaneurysms treated successfully by the open approach have been well-documented in the literature. Shield et al. showed a case of a 54-year-old man who had an iatrogenic thyrocervical trunk pseudoaneurysm repaired by an open approach, without post-operative issues [16]. Similarly, Abrokwah et al. [17] and den Hollander and Slapak [18] described a case of a thyrocervical trunk pseudoaneurysm treated successfully with an open approach. No complications were reported post-operatively.

A limitation of this case report is that we could not get the post-operative DUS imaging for the proper documentation of the pseudoaneurysm resolution.

As seen with our case, open surgical resection is a valid and useful option in treating thyrocervical trunk pseudoaneurysms, if significantly large and/or located at the root of the trunk, which can be potentially difficult to treat via an endovascular approach.

## Conclusions

Thyrocervical trunk pseudoaneurysm is a rare entity usually secondary to trauma or iatrogenic in nature. The incidence of idiopathic thyrocervical trunk pseudoaneurysm is even rarer with limited documentation in the literature. Given the advancement of endovascular technology in recent years, these pseudoaneurysms are frequently treated endovascularly with great results. However, as demonstrated in this case report, a large thyrocervical trunk pseudoaneurysm can be successfully treated with an open approach and without perioperative complications. Therefore, this is a safe, valid, and effective treatment when the pseudoaneurysm is located at the root of the trunk and significantly large, which makes the anatomy of the pseudoaneurysm and its relation with main vessels very complex. An open approach in this situation, when done properly, can be very reliable and with low complication rates.
